# Supplementary material for: ΦCrAss001 represents the most abundant bacteriophage family in the human gut and infects Bacteroides intestinalis
Source: Nat Commun. 2018 Nov 14;9:4781. doi: 10.1038/s41467-018-07225-7 (PMC6235969; doi:10.1038/s41467-018-07225-7)
Supplement: Supplementary file 1 — Supplementary Information [file 41467_2018_7225_MOESM1_ESM.pdf]

Supplementary Figures

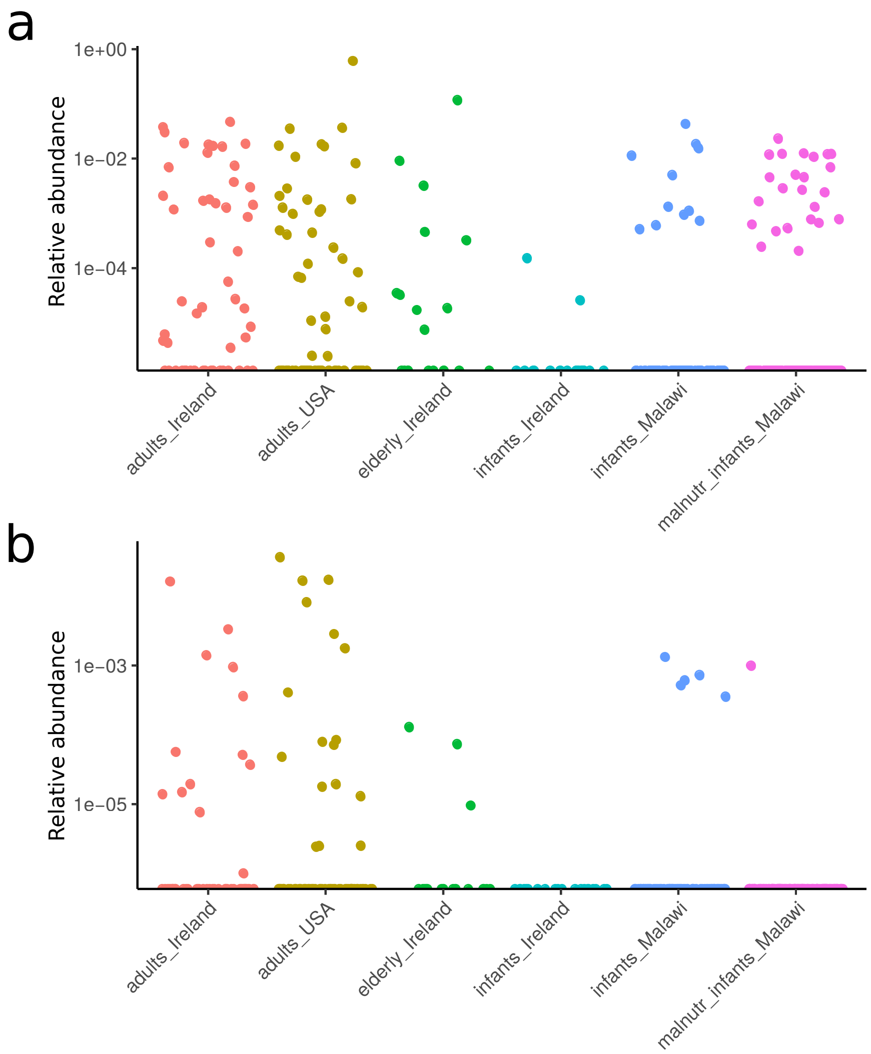

Supplementary Figure 1. Incidence of ΦcrAss001 and related bacteriophages in the human faecal viromes. A, cumulative relative abundance (fraction of reads aligned) of different IAS-virus like phage contigs in several faecal virome metagenomic sequencing datasets[1–3]; **B**, relative abundance of ΦcrAss001 in the same datasets.

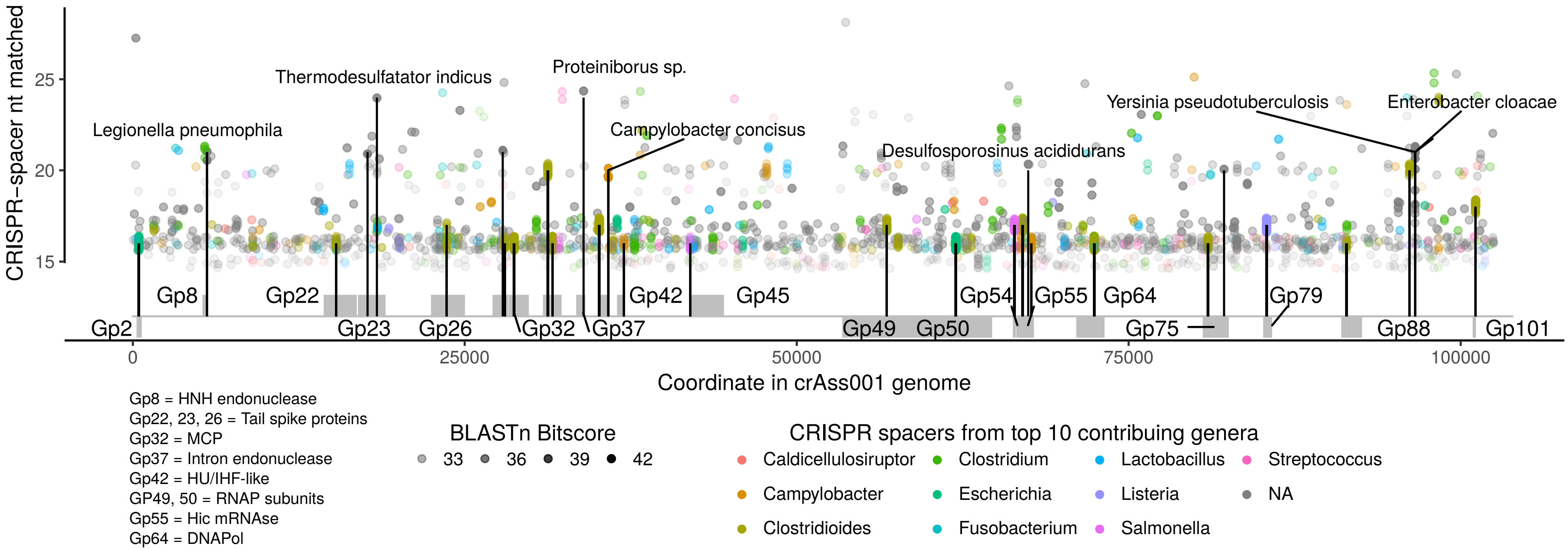

Supplementary Figure 2. Distribution of BLASTn hits of CRISPR spacers from 5,591 bacterial genomes on the ΦcrAss001 genome map. Dots indicate individual CRISPR spacer sequences (best 3000 hits, dot intensity proportional to the BLASTn Bitscore); coloured dots correspond to top 10 contributing bacterial genera; vertical lines highlight either top scoring hits (Bitscore > 20, e-value < 0.001, dubbed with species of origin name) or hotspots with >10 hits at same coordinate; grey blocks represent ORFs in which top-scoring hits or hotspots are located; + strand ORFs are located above the centre line, - strand ORFs are located beneath it.

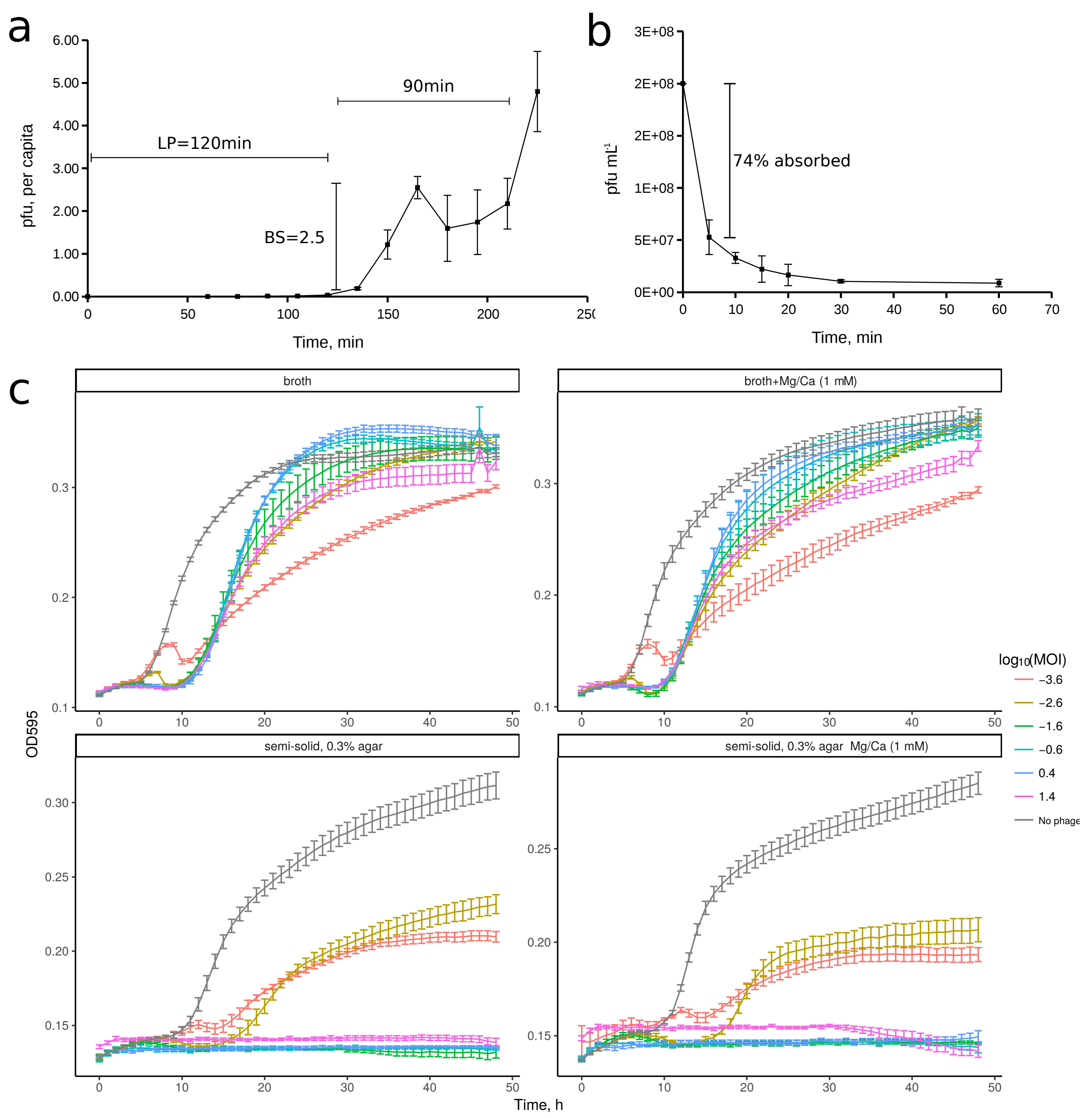

Supplementary Figure 3. Biological properties of ΦcrAss001. A, one-step growth curve of ΦcrAss001 (pfu *per capita*, mean±SD of four biological replicates) in early log-phase culture *B. intestinalis* 919/174 (OD_{600} = 0.2 at inoculation, MOI = 0.7); LP, latency period; BS, burst size (see text for details); **B**, adsorption curve of ΦcrAss001 (pfu/ml, mean±SD of three biological replicates) on early-log phase *B. intestinalis* 919/174 cells in FAB medium (MOI = 0.7); **C**, growth curves of *B. intestinalis* 919/174 (OD_{600}, mean±SD of three biological replicates) infected at the early-log growth phase (OD_{600} = 0.2 at inoculation) with different MOI levels of ΦcrAss001 (MOI ~ 0.00025 – 25).

Supplementary References

1. Manrique, P. *et al.* Healthy human gut phageome. *Proc. Natl. Acad. Sci. U. S. A.* **113,** 10400–10405 (2016).
2. Norman, J. M. *et al.* Disease-specific Alterations in the Enteric Virome in Inflammatory Bowel Disease. *Cell* **160,** 447–460 (2015).
3. Reyes, A. *et al.* Gut DNA viromes of Malawian twins discordant for severe acute malnutrition. *Proc. Natl. Acad. Sci. U. S. A.* **112,** 11941–11946 (2015).
