# Supplementary material for: ΦCrAss001 represents the most abundant bacteriophage family in the human gut and infects Bacteroides intestinalis
Source: Nat Commun. 2018 Nov 14;9:4781. doi: 10.1038/s41467-018-07225-7 (PMC6235969; doi:10.1038/s41467-018-07225-7)
Supplement: Supplementary file 3 — Description of Additional Supplementary Files [file 41467_2018_7225_MOESM3_ESM.pdf]

Description of Additional Supplementary Files

File Name: Supplementary Data 1
Description: List of strains used for enrichment experiments in the isolation of ϕcrAss001

File Name: Supplementary Data 2
Description: HHPRED annotation results for gene products predicted in ϕcrAss001

File Name: Supplementary Data 3
Description: HHPRED annotation results for gene products predicted in contig Fferm_ms_2
