# Supplementary material for: ΦCrAss001 represents the most abundant bacteriophage family in the human gut and infects Bacteroides intestinalis
Source: Nat Commun. 2018 Nov 14;9:4781. doi: 10.1038/s41467-018-07225-7 (PMC6235969; doi:10.1038/s41467-018-07225-7)
Supplement: Supplementary file 7 — Reporting Summary [file 41467_2018_7225_MOESM7_ESM.pdf]

nature research

Corresponding author(s): Colin Hill, Andrey Shkoporov

Reporting Summary

Nature Research wishes to improve the reproducibility of the work that we publish. This form provides structure for consistency and transparency in reporting. For further information on Nature Research policies, see Authors & Referees and the Editorial Policy Checklist.

Statistical parameters

When statistical analyses are reported, confirm that the following items are present in the relevant location (e.g. figure legend, table legend, main text, or Methods section).

n/a	Confirmed	
☐	☒	The exact sample size (n) for each experimental group/condition, given as a discrete number and unit of measurement
☐	☒	An indication of whether measurements were taken from distinct samples or whether the same sample was measured repeatedly
☒	☐	The statistical test(s) used AND whether they are one- or two-sided<br>*Only common tests should be described solely by name; describe more complex techniques in the Methods section.*
☒	☐	A description of all covariates tested
☒	☐	A description of any assumptions or corrections, such as tests of normality and adjustment for multiple comparisons
☒	☐	A full description of the statistics including central tendency (e.g. means) or other basic estimates (e.g. regression coefficient) AND variation (e.g. standard deviation) or associated estimates of uncertainty (e.g. confidence intervals)
☒	☐	For null hypothesis testing, the test statistic (e.g. F, t, r) with confidence intervals, effect sizes, degrees of freedom and P value noted<br>*Give P values as exact values whenever suitable.*
☒	☐	For Bayesian analysis, information on the choice of priors and Markov chain Monte Carlo settings
☒	☐	For hierarchical and complex designs, identification of the appropriate level for tests and full reporting of outcomes
☒	☐	Estimates of effect sizes (e.g. Cohen's d, Pearson's r), indicating how they were calculated
☐	☒	Clearly defined error bars<br>*State explicitly what error bars represent (e.g. SD, SE, CI)*

Our web collection on statistics for biologists may be useful.

Software and code

Policy information about availability of computer code

Data collection	FastQC v0.11.5; Trimmomatic v0.36
Data analysis	metaSPAdes v3.10.0; BLASTn v2.2.28+; Bowtie v2.1.0; Samtools v0.1.19; VIGA v0.10.3; GView v1.7; Easyfig v. 2.2.2; PhageTerm v1.0.10; PILER-CR v1.06; ARAGORN v1.2.36; R 3.4.4 and custom R scripts provided in Source Data Files.

For manuscripts utilizing custom algorithms or software that are central to the research but not yet described in published literature, software must be made available to editors/reviewers upon request. We strongly encourage code deposition in a community repository (e.g. GitHub). See the Nature Research guidelines for submitting code & software for further information.

Data

Policy information about availability of data

All manuscripts must include a data availability statement. This statement should provide the following information, where applicable:

- Accession codes, unique identifiers, or web links for publicly available datasets
- A list of figures that have associated raw data
- A description of any restrictions on data availability

Bioproject PRJNA486819 [https://www.ncbi.nlm.nih.gov/bioproject/?term=PRJNA486819] (bacteriophage crAss001 genomic DNA, raw reads); GenBank MH675552 [https://www.ncbi.nlm.nih.gov/nuccore/MH675552] (bacteriophage crAss001 assembled and annotated genome); GenBank QRES00000000 [https://

www.ncbi.nlm.nih.gov/nuccore/QRES00000000] (bacteriophage host Bacteroides intestinalis APC919/174 assembled and annotated genome). Source data for figures 2 and 3, as well as for supplementary figures 1, 2 and 3 are provided as a Source Data files with the paper.

Field-specific reporting

Please select the best fit for your research. If you are not sure, read the appropriate sections before making your selection.

☒ Life sciences ☐ Behavioural & social sciences ☐ Ecological, evolutionary & environmental sciences

For a reference copy of the document with all sections, see nature.com/authors/policies/ReportingSummary-flat.pdf

Life sciences study design

All studies must disclose on these points even when the disclosure is negative.

Sample size	N/A
Data exclusions	N/A
Replication	All tests were performed in triplicates or quadruplicates and were fully reproducible.
Randomization	N/A
Blinding	N/A

Reporting for specific materials, systems and methods

Materials & experimental systems

n/a	Involved in the study	
☐	☒	Unique biological materials
☒	☐	Antibodies
☒	☐	Eukaryotic cell lines
☒	☐	Palaeontology
☒	☐	Animals and other organisms
☐	☒	Human research participants

Methods

n/a	Involved in the study	
☒	☐	ChIP-seq
☒	☐	Flow cytometry
☒	☐	MRI-based neuroimaging

Unique biological materials

Policy information about availability of materials

Obtaining unique materials	Bacteriophage crAss001 and its host Bacteroides intestinalis APC919/174 are available from the authors.

Human research participants

Policy information about studies involving human research participants

Population characteristics	The study cohort consisted from 20 clinically healthy Irish adults (23-54 years old, both males and females) with no recent history of gastrointestinal diseases, antibiotic or probiotic consumption.
Recruitment	Volunteers among mainly from APC/UCC students and staff.
